# Mammary Diseases in a Captive Reared Agouti (*Dasyprocta leporina*) in Trinidad

**DOI:** 10.3390/vetsci7030137

**Published:** 2020-09-19

**Authors:** Kegan Romelle Jones, Kavita Ranjeeta Lall, Rod Suepaul, Gary Wayne Garcia

**Affiliations:** 1 Department of Basic Veterinary Sciences (DBVS), School of Veterinary Medicine (SVM), University of the West Indies (UWI), Mt. Hope, Trinidad and Tobago; Rod.Suepaul@sta.uwi.edu; 2 Department of Food Production (DFP), Faculty of Food and Agriculture (FFA), University of the West Indies (UWI), St. Augustine, Trinidad and Tobago; k_lee_24@yahoo.com (K.R.L.); prof.gary.garcia@gmail.com (G.W.G.)

**Keywords:** *Staphylococcus aureus*, mammary gland hyperplasia, wildlife farmers, infectious diseases

## Abstract

The agouti (*Dasyprocta leporina*) is a neotropical rodent that is utilized for its meat by hunters as well as wildlife farmers. There is a dearth of information on infectious diseases that affect these animals. At present, there has been no recording in the literature on diseases of mammary tissue in these animals. This case reported on the abnormal mammary enlargement of a four year old female agouti post-partum. Blood, milk and tissue samples were taken for diagnostics to determine the cause of disease. Histological samples confirmed the swelling of the mammary gland as a diffuse mammary hyperplasia. Hematological values obtained were within the reference range of agoutis reared in captivity. The milk samples that were taken cultured *Staphylococcus* spp. in one mammary gland (left inguinal). The cultured bacteria in the milk samples confirmed this animal had mastitis. The bacterial cultured (*Staphylococcus aureus*) was sensitive to tetracyclines, ampicillin, trivetrin and ceftiofur. To the authors’ knowledge, this is the first record in the literature on mastitis in the agouti. Thus, this information will add to the knowledge of diseases in captive reared agoutis.

## 1. Introduction

The agouti (*Dasyprocta leporina*) is a rodent found in the Neotropics [1]. This animal is one of the most hunted animal species in South America [2]. It is utilized for its meat and the agouti provides food security to rural communities. Some authors have identified the agouti as one of six neotropical mammalian species that has the potential for domestication [3]. Hardiun et al. [4] grouped the agouti as a mini-livestock animal that can be farm reared as a source of meat protein in developing countries.

With increased hunting pressure of the wild population of the agouti, rearing these animals in captivity becomes a viable ex-situ conservation strategy. In the quest to domesticate and operate intensive farming systems of the agouti, information on the health of the animal must be known. In the agouti literature, there is information on the infectious agents that cause disease. Gastrointestinal parasites such as *Trichuris* spp., *Strongyloides* spp. and *Paraspidodera uncinata* have been identified in captive and wild populations of the agouti [5,6,7,8,9,10,11]. Blood parasites such as *Babesia* spp. and *Trypanosoma* spp. have also been found in wild agoutis [12,13] but not in captive reared animals [14]. Lall et al. [15] further summarized infectious agents that infected the agouti but found few cases of clinical diseases due to these agents.

In contrast to infectious diseases, there is little information on non-infectious disease that affects the agouti. There have been some reports on Vitamin D toxicity [16,17], dystocia [18] and uterine tumors [19]. Some non-infectious diseases of captive reared agoutis which were diagnosed by pathological examination include: esophageal obstruction, cecal and fecal impaction, urolithiasis, polycystic kidneys, fetal maceration and mummification, dystocia, retained placenta, endometritis and pyometra [20,21,22]. However, in the literature there was no information of diseases that affect the mammary tissue of the agouti. Therefore, this is the first recorded case of mastitis and secondary mammary hyperplasia in the agouti. The objective of this report is to report the cause of the enlarged mammary tissue in a captive reared female agouti.

## 2. Materials and Method

### 2.1. Ethical Approval

All applicable international, national, and/or institutional guidelines for the care and use of animals were followed. The research site has been overseen by veterinarians to ensure animals are kept healthy. Field and laboratory protocols were approved by the Ethics Committee of the University of the West Indies, Faculty of Food and Agriculture, University of the West Indies, St. Augustine campus (ref no. CEC446/04/19).

### 2.2. Housing and Environment of the Agouti

The agouti was located on the University Field Station farm at the Neotropical animal unit at Mount Hope Trinidad. At this location, animals were grown in both caged and floored systems. The floor rearing system was used for breeding animals, while the cage-reared system was used for housing pregnant female animals as well as the replacement male animals. Animals were fed with local feedstuffs that were found on the farm. These included banana (*Musa* spp.), pumpkin (*Cucurbita pepo*), mangoes (*Mangifera indica*), trichantera (*Trichanthera gigantea*) and papaya (*Carica papaya*). Agoutis were supplemented with chicken (*Gallus domesticus*) eggs and pelleted rabbit ration (Mastermix^®^).

### 2.3. History and Physical Examination

An adult four-year-old female agouti was observed to have abnormally enlarged mammary glands at parturition. The animal was bright, alert and responsive, the mammary glands felt firm on palpation and she gave birth to a single offspring. The offspring died two days after parturition, as it was unable to suckle due to the enlargement of the mammary glands.

### 2.4. Haematological and Serum Biochemical Test

The animal was sedated with xylazine 5 mg/kg and ketamine at 5 mg/kg intramuscularly prior to collection of blood samples and analysis was done at the School of Veterinary Medicine, University of the West Indies. Blood was taken from the saphenous vein using a 22-gauge 1-inch need. Approximately 4 mL of blood was taken from the animal. Two mL of blood was placed in a red top tube (No anticoagulant, Medisure Ltd. Florida, USA) for serum biochemistry while the other 2 mL of blood was placed in a purple top tube (EDTA tube, Medisure Ltd.) from analysis of the blood cells.

### 2.5. Milk Sample Test

Each teat was cleaned with a disinfectant solution and dried before milk samples were collected. Milk was collected from each mammary gland and placed in separate red top tubes. The milk samples were tested for the presence of bacteria using bacterial culture and antibiotic sensitivity techniques. Milk samples were placed on blood agar plates for 24 h at 37 °C in an aerobic environment. The blood agar was made using blood from sheep that were reared at the School of Veterinary Medicine, University of the West Indies. The colonies were then identified using morphological and biochemical testing. The colonies were stained with Gram’s stain and observed under light microscopy. The color and general appearance on blood agar was recorded. Bacterial colonies were also analyzed using biochemical tests such as catalase and tube coagulase tests. Finally, the colonies were analyzed for antimicrobial sensitivity for tetracyclines, ampicillin, trivetrin and ceftiofur. Milk samples were analyzed in the Bacteriology Lab at the School of Veterinary Medicine, University of the West Indies.

### 2.6. Tissue Biopsy and Histology

Tissue samples of the mammary gland were taken from the sedated agouti. The samples were removed surgically, and the wound was closed using 3-0 vicryl (Ethicon^®^, NJ, USA). The tissue sample that was taken was placed into a container with 10% formalin. The tissue samples were stained using Hematoxylin Eosin (HE) stain. The histopathology test on the sample was done at the School of Veterinary Medicine, University of the West Indies.

## 3. Results

### 3.1. Gross Findings

The animal was bright, alert and responsive. The mammary glands of the animal were enlarged and felt firm on palpating. All eight mammary glands were enlarged, and the offspring was unable to suckle due to the size of the mammary glands and pain due to suckling. The offspring subsequently died due to undernutrition. The agouti was not treated with any form of antibiotic drugs and the mammary swellings resolved after three months.

### 3.2. Blood and Milk Samples Findings

Bacterial colonies that were grown from the blood agar plates for 24 h in an aerobic environment at 37 °C were cream and pinpoint. After the colonies were Gram stained they appeared as Gram-positive cocci in clusters under light microscopy. The colonies were also catalase positive and coagulase positive. The colonies produced hemolysis on the blood agar plate. These finding (morphological and biochemical) identified the bacteria as a *Staphylococcus* spp. but more specifically due to the appearance, biochemical tests (catalase and coagulase test) and the hemolysis on blood agar as *S. aureus* [23,24] (Table 1). The cultured bacteria were sensitive to tetracyclines, ampicillin, trivetrin and ceftiofur. The cultured sample was obtained from the left inguinal mammary gland. The samples from the remaining mammary glands were not diagnostic. The complete blood count (CBC) for the animal showed no abnormalities and all values were within reference ranges for agoutis reared in captivity [14,25,26,27].

### 3.3. Histopathological Findings

**Figure 1:**
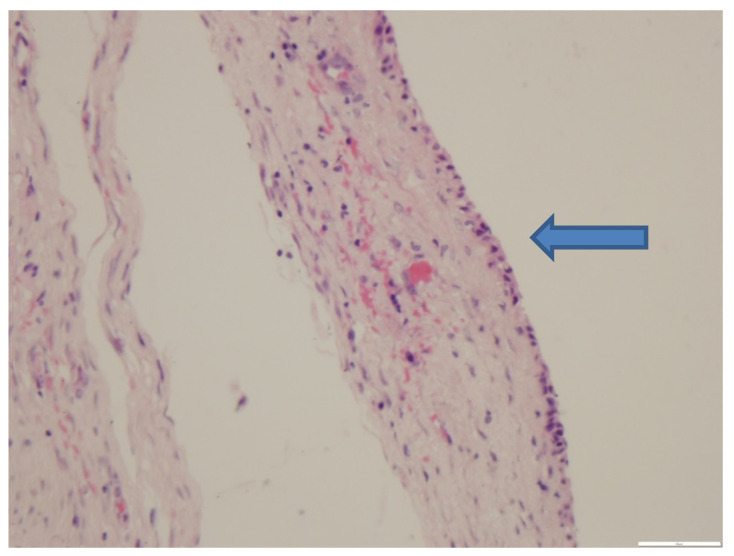
The thick fibrous capsule and the attenuated cuboidal epithelium (arrow). HE stain, 40× magnification.

The mass had a thick fibrous capsule with the center filled with foamy eosinophilic proteinaceous material and lined by a single layer of attenuated cuboidal epithelium (Figure 1). At the edges were multiple foamy macrophages and multinucleated giant cells variably containing cholesterol clefts. The contents of the structure, the epithelial lining and the cholesterol clefts indicate the presence of a mammary gland hyperplasia. The cuboidal epithelium and eosinophilic contents with cholesterol clefts as well as location on the animal strongly suggest mammary origin.

## 4. Discussion

Pathology of the mammary glands of the agouti has never been recorded in the literature. The agouti (*D. leporina*) has four pairs of mammary teats; one cranial teat pair, two abdominal teat pairs and one caudal teat pair. In captivity, all eight mammary teats were reported to be functional with females producing 1–3 offspring per parturition [3,28]. In this study, the agouti produced one offspring that died within a week after parturition. It can be assumed that the cause of death was due to starvation as the offspring was not able to suckle due to the enlarged mammary glands. The mortality of the offspring in this case contrasts with information on mastitis in guinea pigs. In guinea pigs, mastitis has no effect on offspring survivability [29].

Analysis of blood samples was within normal reference ranges for captive reared *Dasyprocta leporina* [25,26,27] The milk samples collected from the mammary glands were positive for *Staphylococcus aureus* which is sensitive to tetracyclines, ampicillin, trivetrin and ceftiofur. This proved that the animal had bacterial mastitis. In other rodent species, mastitis has been caused by other bacterial pathogens. In rats, *Pasteurella pneumotropica*, *Pseudomonas aeruginosa*, *Escherichia coli* and *Staphylococcus aureus* have been isolated from swollen and discolored mammary glands [30]. In guinea pigs, α-hemolytic *Streptococcus* was identified in a breeding colony. The milk had a viscosity similar to that of blood upon collection and the mammary glands were swollen, conjected and indurated the following day [31]. α-hemolytic *Streptococcus* in the milk samples were sensitive to many antibiotics (chlortetracycline, bacitracin, chloramphenicol, nitrofurazone, lincomycin, penicillin, erythromycin, dihydrostreptomycin, neomycin, polymyxin B and oxytetracyclines) which was also seen in this case [31].

Furthermore, Kinkler et al. [29] reported on bacterial mastitis cases in guinea pigs. It was found that spontaneous mastitis followed two clinical courses. Clinical signs seen in the first course were inappetence, dehydration, depression, oligodipsia and weight loss. The mammary glands were generally swollen and had red to purple discolorations. Animals within this course usually died shortly after clinical signs. In the second course, a slight increase in the size of the mammary gland was seen without discoloration. The information provided by Kinkler et al. [29] would suggest that the agouti suffered from the second clinical course. In the second or the chronic form, mammary glands are not discolored, and general clinical signs are absent.

Histology also confirmed the animal had mastitis by the presence of foamy macrophages and multi-nucleated giant cells. The enlarged mammary gland had a fibrous capsule with eosinophilic proteinaceous material. The histological results implied that the enlarged glands (mammary hyperplasia) were due to the mastitis. In rats, affected glands showed massive necrosis of the parenchyma and accumulation of neutrophils, lymphocytes and few giant cells [30]. In some cases, as was seen in breeding rats, bacteria can be identified extracellularly as well as intracellularly (within neutrophils) [30]. In the guinea pig, mastitis was diffused with lobular necrosis and vacuolar degeneration. The necrotic foci were surrounded by neutrophils and immature fibroblasts. The interalveolar spaces were also proliferated with neutrophils and plasma cells [31]. Further to this, mastitis in guinea pigs was categorized as acute and chronic forms of the disease. In the acute form, the mammary gland is congested, hemorrhagic and edematous. There is degeneration and sloughing of alveolar epithelial cells, polymorphonuclear leukocytes and proteinaceous basophilic material fill the alveolar lumina. The chronic syndrome showed the primary chronic inflammatory response with moderate interalveolar fibroplasia [29]. In the case of the agouti, it can be deduced that it was the chronic form of the disease based on clinical signs and histopathological findings.

Mastitis in rabbits was reported to be caused by *S. aureus* and some work on the various biotypes has been conducted [32]. However, the biotypes found in mammary glands were not similar to those that inhabited the surfaces of the skin. This shows that in rabbits, *S. aureus* has biotypes with variations in virulence. Further work was done to identify four histopathological categories (abscessation, suppurative mastitis, cellulitis and mixed lesions) of mastitis in rabbits. The author noted that there was no correlation between the category of chronic mastitis and the genotypes of *S. aureus* [33]. More recently, mammary glands of rabbits that were infect with *S. aureus* ST96 strain had fewer granulocytes, greater B cells, T cells, CD^+^4 T cells and CD^+^8 T cells, compared to mammary glands infected with ST 121 strains [34]. In the agouti in this case, *Staphylococcus aureus* was identified to have caused inflammation of the mammary gland. Further work can be done to investigate the composition of the normal flora of the skin as well as the bacterial content of milk samples from asymptomatic agoutis.

## 5. Conclusions

This study examined the abnormal mammary swellings of a four-year-old female agouti reared in captivity. Bacterial culture of the milk samples identified *Staphylococcus aureus*. This proved that the agouti was suffering from bacterial mastitis. Histological examination of the mammary tissue showed the animals also had diffuse mammary hyperplasia due to the mammary infection. To the authors’ knowledge, information on the mammary pathology in the agouti was never reported and will add to understanding of the diseases these animals can experience in captivity.

## Figures and Tables

**Table 1:** Morphological, coagulase and catalase tests done on milk samples from the Agouti (*D. leporina*).

Morphological Characteristics/Tests	Results
Gram Stain	Gram (+) cocci
Catalase Test	Catalase (+)
Appearance on Blood Agar	Cream pinpoint colonies with zones of hemolysis
Coagulase test	Coagulase (+)
